# A Structure-Function Diversity Survey of the RNA-Dependent RNA Polymerases From the Positive-Strand RNA Viruses

**DOI:** 10.3389/fmicb.2019.01945

**Published:** 2019-08-22

**Authors:** Hengxia Jia, Peng Gong

**Affiliations:** ^1^Key Laboratory of Special Pathogens and Biosafety, Center for Biosafety Mega-Science, Wuhan Institute of Virology, Chinese Academy of Sciences, Wuhan, China; ^2^University of Chinese Academy of Sciences, Beijing, China

**Keywords:** positive-strand RNA virus, RNA-dependent RNA polymerase, genome replication, structure, catalytic motif

## Abstract

The RNA-dependent RNA polymerases (RdRPs) encoded by the RNA viruses are a unique class of nucleic acid polymerases. Each viral RdRP contains a 500–600 residue catalytic module with palm, fingers, and thumb domains forming an encircled human right hand architecture. Seven polymerase catalytic motifs are located in the RdRP palm and fingers domains, comprising the most conserved parts of the RdRP and are responsible for the RNA-only specificity in catalysis. Functional regions are often found fused to the RdRP catalytic module, resulting in a high level of diversity in RdRP global structure and regulatory mechanism. In this review, we surveyed all 46 RdRP-sequence available virus families of the positive-strand RNA viruses listed in the 2018b collection of the International Committee on Virus Taxonomy (ICTV) and chose a total of 49 RdRPs as representatives. By locating hallmark residues in RdRP catalytic motifs and by referencing structural and functional information in the literature, we were able to estimate the N- and C-terminal boundaries of the catalytic module in these RdRPs, which in turn serve as reference points to predict additional functional regions beyond the catalytic module. Interestingly, a large number of virus families may have additional regions fused to the RdRP N-terminus, while only a few of them have such regions on the C-terminal side of the RdRP. The current knowledge on these additional regions, either in three-dimensional (3D) structure or in function, is quite limited. In the five RdRP-structure available virus families in the positive-strand RNA viruses, only the *Flaviviridae* family has the 3D structural information resolved for such regions. Hence, future efforts to solve full-length RdRP structures containing these regions and to dissect the functional contribution of them are necessary to improve the overall understanding of the RdRP proteins as an evolutionarily integrated group, and our analyses here may serve as a guideline for selecting representative RdRP systems in these studies.

## Introduction

First identified in the 1950s in the mengovirus and poliovirus (PV) related studies (Reich et al., 1961, 1962), the RNA-dependent RNA polymerases (RdRPs) encoded by the RNA viruses catalyze the RNA synthesis from RNA templates, and are responsible for the viral genome replication and transcription processes. As the essential and the most conserved protein from the RNA viruses, the RdRPs are attractive systems both for understanding the fundamentals of nucleic acid synthesis and for developing antiviral strategies. Each RdRP contains a catalytic module (or catalytic core) with an overall architecture resembling an encircled human right hand that is composed of the palm, fingers, and thumb domains (Hansen et al., 1997; Ago et al., 1999; Bressanelli et al., 1999; Lesburg et al., 1999). The size of the catalytic module is typically about 50 and 70 kilo-Dalton (kD) for primer-dependent and *de novo* RdRPs, respectively, regarding the RdRP initiation mode (Lesburg et al., 1999; Thompson and Peersen, 2004). However, the size of the RdRP protein could reach 240–450 kD (Kinsella et al., 2004; Liang et al., 2015; Gerlach et al., 2015), often due to the requirement of incorporating other functional modules or as a result of coevolution with different host species. To some extent, the conservation of the catalytic module and the diversity of the full-length RdRP protein are two important aspects in understanding this unique class of polymerases. In this review, we surveyed all 46 RdRP-sequence available virus families listed in the International Committee on Virus Taxonomy (ICTV)^1^ 2018b collection from the positive-strand RNA virus category [ssRNA(+) in the ICTV genome composition assignment] and tend to provide an RdRP reference map based on known information of RdRP structure and function. The main purpose of this work is to place the current knowledge of these RdRPs in a broader context and to facilitate future studies in RdRPs with representative primary structure and/or with distinct functions beyond the catalytic module, thereby help improve the overall understanding of RdRP structure, function, and evolution. Based on sequence availability in the United States National Center for Biotechnology Information (NCBI) database^2^, one representative RdRP amino acid sequence was chosen for each virus family by giving the priority to the ICTV-suggested type species (Table 1). As an exception, one representative sequence was chosen for each virus genus for the *Flaviviridae* family, since very different RdRPs primary structure has been identified in this virus family. For simplicity, we used residues in the PV RdRP (also known as the 3D^pol^ protein) to define conserved sites.

**TABLE 1:** Virus taxonomy assignments, virus name abbreviations, GenBank accession numbers, and related Protein Data Bank (PDB) entries of the representative positive-strand RNA virus RdRPs chosen for analyses in this study.

**Virus species**	**Genus^a^**	**Family^b^**	**Order^c^**	**Abbreviation**	**GenBank Acc. no.**	**PDB^d^**
*Equine arteritis virus*	*Alphaarteri-*	*Arteri-*	*Nido-*	EAV (van Dinten et al., 1999; Ziebuhr et al., 2000)	NC_002532	
*Severe acute respiratory syndrome-related coronavirus*^e^	*Betacorona-*	*Corona-*	*Nido-*	SARS-CoV (Snijder et al., 2003; Subissi et al., 2014a)	NC_004718	
*Aplysia abyssovirus 1*	*Alphaabysso-*	*Abysso-*	*Nido-*	AAbV	GBBW01007738	
*Charybnivirus 1*	*Charybni-*	*Euroni-*	*Nido-*	CharNV	KX883628	
*Turrinivirus 1*	*Turrini-*	*Medioni-*	*Nido-*	TurrNV	KX883629	
*Alphamesonivirus 1*	*Alphamesoni-*	*Mesoni-*	*Nido-*	NDiV	MH520106	
*Planarian secretory cell nidovirus*	*Alphamononi-*	*Mononi-*	*Nido-*	PSCNV	MH933735	
*Gill-associated virus*	*Oka-*	*Roni-*	*Nido-*	GAV	NC_010306	
*White bream virus*	*Bafini-*	*Tobani-*	*Nido-*	WBV	NC_008516	
*Israel acute paralysis virus*^e^	*Apara-*	*Dicistro-*	*Picorna-*	IAPV (de Miranda et al., 2010)	NC_009025	
*Infectious flacherie virus*	*Ifla-*	*Ifla-*	*Picorna-*	IFV (Isawa et al., 1998)	AB000906	
*Poliovirus 1*	*Entero-*	*Picorna-*	*Picorna-*	PV-1 (Harris et al., 1992)	NC_002058	1RA6
*Cowpea mosaic virus*	*Como-*	*Seco-*	*Picorna-*	CPMV (Peters et al., 1995)	X00206	
*Solenopsis invicta virus 2*	*Sopolyci-*	*Polycipi-*	*Picorna-*	SINV-2	EF428566	
*Heterosigma akashiwo RNA virus*	*Marna-*	*Marna-*	*Picorna-*	HaRNAV	NC_005281	
*Turnip yellow mosaic virus*	*Tymo-*	*Tymo-*	*Tymo-*	TYMV (Morch et al., 1988; Jakubiec et al., 2007; Moriceau et al., 2017)	NC_004063	
*Potato virus X*	*Potex-*	*Alphaflexi-*	*Tymo-*	PVX	NC_011620	
*Grapevine virus A*	*Viti-*	*Betaflexi-*	*Tymo-*	GVA	AF007415	
*Sclerotinia sclerotiorum deltaflexivirus 1*	*Deltaflexi-*	*Deltaflexi-*	*Tymo-*	SsDFV1	NC_038977	
*Botrytis virus F*	*Mycoflexi-*	*Gammaflexi-*	*Tymo-*	BotV-F	NC_002604	
*Nudaurelia capensis beta virus*	*Betatetra-*	*Alphatetra-*	/^f^	NβV (Gorbalenya et al., 2002)	NC_001990	
*Human astrovirus 8*	*Mamastro-*	*Astro-*	/	HAstV-8 (Willcocks et al., 1994; Mendez-Toss et al., 2000)	AF260508	
*Mushroom bacilliform virus*	*Barna-*	*Barna-*	/	MBV (Revill et al., 1994)	NC_001633	
*Beet necrotic yellow vein virus*	*Beny-*	*Beny-*	/	BNYVV (Hehn et al., 1997)	NC_003514	
*Ourmia melon virus*	*Ourmia-*	*Botourmia-*	/	OuMV (Rastgou et al., 2009)	EU770623	
*Brome mosaic virus*	*Bromo-*	*Bromo-*	/	BMV (Ahlquist et al., 1984)	NC_002027	
*Norwalk virus*	*Noro-*	*Calici-*	/	NV (Ng et al., 2004; Oliver et al., 2006)	AJ583672	1SH0
*Providence virus*	*Alphacarmotetra-*	*Carmotetra-*	/	PrV (Walter et al., 2010)	NC_014126	
*Lettuce infectious yellows virus*	*Crini-*	*Clostero-*	/	LIYV (Klaassen et al., 1995)	U15440	
*Yellow fever virus*	*Flavi-*	*Flavi-*	/	YFV (Rice et al., 1985)	X03700	4K6M
*Hepatitis C virus*	*Hepaci-*	*Flavi-*	/	HCV (Yanagi et al., 1997)	AF011751	1C2P
*Hepatitis G virus*^e^	*Pegi-*	*Flavi-*	/	HGV (Xiang et al., 2000; Berg et al., 2015)	NC_001710	
*Bovine viral diarrhea virus 1*	*Pesti-*	*Flavi-*	/	BVDV (Choi et al., 2004)	NC_001461	5YF5
*Cutthroat trout virus*	*Piscihepe-*	*Hepe-*	/	CTV (Batts et al., 2011)	NC_015521	
*Blueberry necrotic ring blotch virus*	*Bluner-*	*Kita-*	/	BNRBV (Quito-Avila et al., 2013)	JN651149	
*Escherichia virus Qbeta*	*Allolevi-*	*Levi-*	/	Qβ (Kidmose et al., 2010)	AWN02713	3MMP
*Barley yellow dwarf virus PAV*	*Luteo-*	*Luteo-*	/	BYDV-PAV (Miller et al., 1988)	NC_004750	
*Saccharomyces 20S RNA narnavirus*	*Narna-*	*Narna-*	/	ScNV-20S (Rodriguez-Cousino et al., 1991, 1998)	NC_004051	
*Nodamura virus*	*Alphanoda-*	*Noda-*	/	NoV (Johnson et al., 2003)	AF174533	
*Thosea asigna virus*	*Alphapermuto tetra-*	*Permutotetra-*	/	TaV (Gorbalenya et al., 2002; Ferrero et al., 2015)	AF282930	4XHI
*Potato virus Y*	*Poty-*	*Poty-*	/	PVY (Singh and Singh, 1996)	U09509	
*Southern bean mosaic virus*	*Sobemo-*	*Solemo-*	/	SBMV (Ozato Junior et al., 2009)	NC_004060	
*Solenopsis invicta virus 3*	*Invicta-*	*Solinvi-*	/	SINV-3 (Valles et al., 2016)	FJ528584	
*Sindbis virus*	*Alpha-*	*Toga-*	/	SINV (Strauss et al., 1984)	J02363	
*Tomato bushy stunt virus*	*Tombus-*	*Tombus-*	/	TBSV (Hearne et al., 1990)	NC_001554	
*Gentian ovary ringspot virus*	*Gora-*	*Virga-*	/	GORV (Atsumi et al., 2015)	NC_024501	
*Heterocapsa circularisquama RNA virus 01*	*Dinorna-*	*Alverna-*	/	HcRNAV01	NC_007518	
*Rubella virus*	*Rubi-*	*Matona-*	/	RUB	RUBCG	
*Cryphonectria hypovirus 3*^e^	*Hypo-*	*Hypo-*	/	CHV3	AF188515	

*^*a*–*c*^ “-” indicates the omitted suffix “virus,” “viridae,” or “virales,” respectively. ^*d*^PDB entries chosen in Figure 3 for seven RdRP-structure available virus families. ^*e*^Non-type species. “/” indicates that virus order has not been assigned. References listed in the abbreviation column are used to define the N- and C-terminal boundaries of the RdRP proteins.*

## Conserved Catalytic Motifs and Residues as Reference Points for Defining the Boundaries of the RdRP Catalytic Module

**FIGURE 1:**
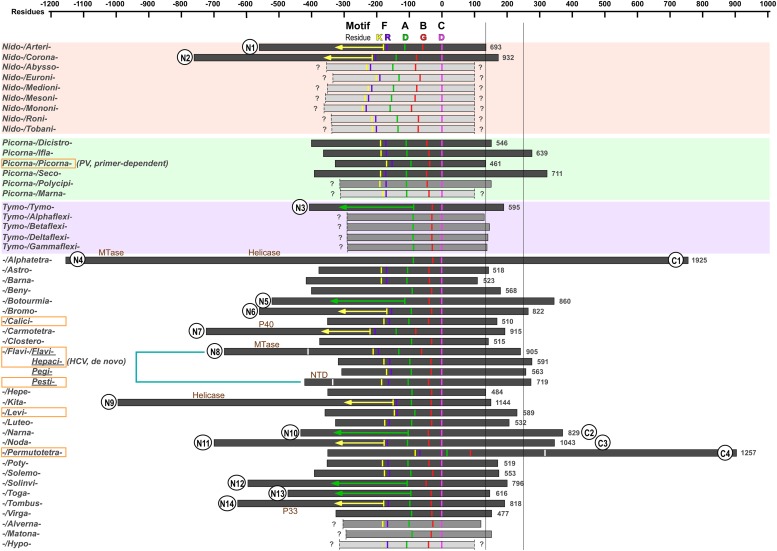
The primary structure comparison of RNA-dependent RNA polymerases (RdRPs) from representative positive-strand RNA viruses. Orders, families, genera, and species assignments based on the ICTV 2018b collection are listed in Table 1. The virus species names are listed in alphabetical order giving the priority to virus order, then to virus family, and then to virus genus. The conserved motif C aspartic acid (magenta, corresponding to PV RdRP D328) is used as the origin in the scale bar. Conserved residues in motifs A, B, and F are also labeled: motif F lysine (corresponding to PV RdRP K159) in yellow; motif F arginine (corresponding to PV RdRP R174) in purple; motif A aspartic acid (corresponding to PV RdRP D233) in green; motif B glycine (corresponding to PV RdRP G289) in red. The orange rectangle indicates that 3D structures are available in that virus family (or virus genus in case of the *Flaviviridae*). The boundaries of the RdRP catalytic module defined by reported 3D structures are indicated by the white bars for the *Flaviviridae* and *Permutotetraviridae* RdRPs. Numbers on the right side of individual RdRP indicate the amino acids numbers for the full-length RdRPs. The question mark (?) indicates undefined boundaries of the RdRP proteins. The yellow and green arrows (150 and 230 residues in length, respectively) are used to estimate the N-terminal boundary of the RdRPs. The two long vertical bars (130 and 250 residues to the origin) indicate the C-terminal boundary of the RdRP catalytic module of the primer-dependent PV 3D^pol^ and the *de novo* HCV NS5B and are used to help predict additional functional regions. Wherever available in literature, the name of additional functional regions are labeled. Circles placed at the RdRP termini indicate predicted additional regions. The numbers following the “N” and “C” simply refer to the number of families possibly having additional regions at the RdRP N- and C-termini, respectively.

A large number of RdRPs from the positive-strand RNA viruses are proteolytic products of viral polyproteins (Palmenberg, 1990; Wimmer and Nomoto, 1993; Reed and Rice, 2000; Bartenschlager et al., 2010; Pietila et al., 2017). Since not all related proteolytic cleavage sites have been reported for some of the virus families, we were only able to define N- and C-terminal boundaries for 33 RdRPs among the 49 representatives (Figure 1 and Table 1). For the rest of the RdRPs, 7 of them only have a defined C-terminus, and 9 of them have both termini undefined based on our best knowledge. Hence, functional studies to identify polyprotein proteolytic sites are necessary to improve the global picture RdRP primary structure diversity, and our analyses are based on incomplete boundary assignments. The overall size of the RdRPs with clear boundaries ranges from ∼460 to ∼1930 residues, indicating that the primary structure of these RdRPs are quite diverse and potential functional regions are likely integrated into some of these RdRP proteins.

**FIGURE 2:**
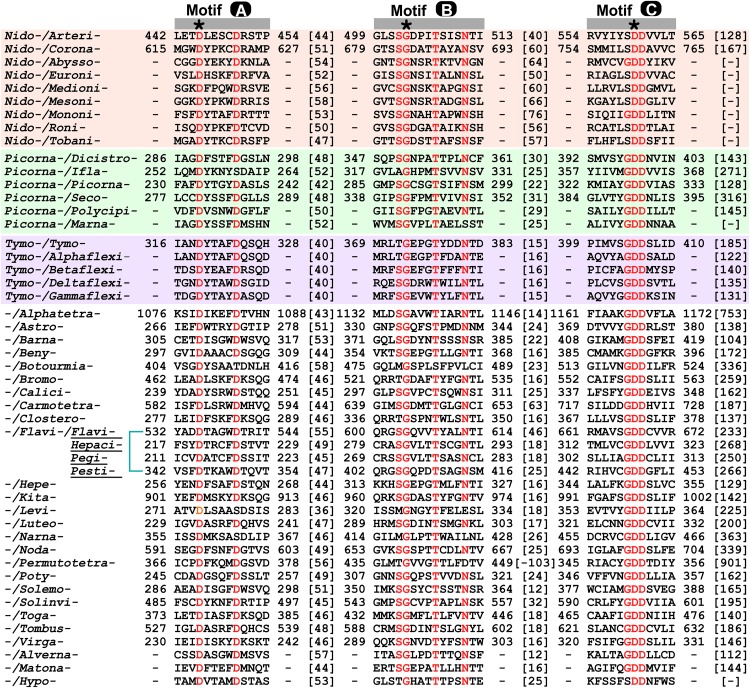
Sequence alignment of motifs A-C of RdRPs from representative positive-strand RNA viruses. The order of motif sequences from top to bottom are consistent with that in Figure 1 and Table 1. Highly conserved residues, including three absolutely conserved residues (labeled by asterisks), are shown in red. Numbers within the brackets indicate the number of residues not shown.

In order to assign or identify possible functional regions beyond the RdRP catalytic module, we use the RdRP catalytic motifs and highly conserved residues to help estimate the boundaries of the catalytic module. The RdRP active site is surrounded by the palm, fingers, and thumb domains with seven catalytic motifs (motifs A–G) distributed within the palm (motifs A–E) and fingers (motifs F–G) (Poch et al., 1989; Gorbalenya et al., 2002; Bruenn, 2003; te Velthuis, 2014; Wu et al., 2015) (see an alignment of motif A–C of the 49 representative RdRP sequences in Figure 2). RdRPs share motifs A/C/D with DNA-dependent polymerases and A–F with the reverse transcriptases (RTs, RNA-dependent DNA polymerases) (Poch et al., 1989; Delarue et al., 1990; Gong and Peersen, 2010), while motif G is an RdRP hallmark motif that may participate in RNA template binding and post-catalysis RdRP translocation on the template (Gorbalenya et al., 2002; Shu and Gong, 2016). Although each catalytic motifs may be well conserved at the levels of virus genus and family, highly conserved residues across different virus families can only be identified in motifs A/B/C/F, and only three residues are absolutely conserved (Figure 2). Among these three residues, two aspartic acid residues in motifs A and C (corresponding to the PV RdRP residues D233 and D328) participate in the coordination interactions with the two divalent metal ions essential for the phosphoryl transfer reaction, and are also required for other classes of polymerases (Beese and Steitz, 1991; Huang et al., 1998; Li et al., 1998; Yin and Steitz, 2004; Zamyatkin et al., 2008; Gong and Peersen, 2010; Appleby et al., 2015). The third absolutely conserved residue is a glycine (corresponding to the PV RdRP residue G289) in motif B. This residue is typically adjacent to an serine and this SG dipeptide plays essential roles in recognizing the 2′-hydroxyl group of the nucleotide triphosphate (NTP) substrate, while the corresponding peptide bond flip accompanies a subtle conformational change of the NTP-induced RdRP active site closure identified by crystallography (Gong and Peersen, 2010; Appleby et al., 2015; Shu and Gong, 2016). It has also been suggested that this glycine residue may be essential for a 3-Å tip movement of the motif B loop (corresponding to the PV RdRP residues 288–292) that could participate in the aforementioned post-catalysis RdRP translocation during each nucleotide addition cycle (NAC) (Sholders and Peersen, 2014). Under either situation, the backbone flexibility of this glycine residue likely explains its requirement at this position. In some cases, the serine residue is replaced by a threonine or even rarely by other residues, and the threonine substitution can also be found in nucleoside analog drug-resistant virus stains (Figure 2; Dutartre et al., 2006; Lam et al., 2012; Flint et al., 2014), suggesting that the side-chain hydroxyl group is the core conservative part of this residue. Motif F typically contains several basic residues and is known to interact with the triphosphate and base moieties of the NTP substrate. Among these residues, one lysine and one arginine (corresponding to the PV RdRP residues K159 and R174) have the highest conservation level (Bruenn, 2003). Hence, we use the absolutely conserved motif C aspartic acid (the first D in the signature sequence XGDD) as the reference point to align all RdRP sequences and use the aforementioned conserved residues to label motifs A/B/C/F (Figure 1). In this way, the relative spacing of these key motifs can be compared in all representative sequences. Typically, the seven motifs appear in the order of G-F-A-B-C-D-E and follow the same protein folding topology. Very interestingly, the RdRPs from the *Permutotetraviridae* family has a different motif order of G-F-C-A-B-D-E. While its spatial organization of the motifs is consistent with that of other RdRPs, the folding topology is permutated (Gorbalenya et al., 2002; Ferrero et al., 2015). A similar situation was found in RdRPs from the *Birnaviridae* family in the double-stranded (ds) RNA virus category (Gorbalenya et al., 2002; Pan et al., 2007). These exceptions suggest that the swapping of the motifs could occur during protein evolution, while the catalytic function could remain largely unaffected. Besides the similarity in the order of RdRP catalytic motifs between the *Birnaviridae* and the *Permutotetraviridae*, the *Birnaviridae* viruses also use a VPg (viral protein genome linked)-mediated initiation mechanism for genome replication and a polyprotein coding strategy that are often found in the positive-strand RNA viruses (Lee et al., 1977; Pan et al., 2007). These observations suggest that the evolutionary boundary between the positive-strand and ds RNA viruses are not definite.

Next, we use two representative RdRPs, the PV 3D^pol^ and the hepatitis C virus (HCV) NS5B to help estimate the boundaries of the catalytic module using conserved residues in motifs A/B/C/F as the reference points (Figure 1). The first reason for choosing these two representatives is that these two proteins are known not to contain functional regions beyond the catalytic module except that the NS5B protein has a 21-residue membrane anchor at its C-terminus (Schmidt-Mende et al., 2001). The second reason is that 3D^pol^ and NS5B represent RdRPs that utilize primer-dependent and *de novo* mechanisms to initiate the RNA synthesis, respectively (Wimmer and Nomoto, 1993; Zhong et al., 2000). Both of these proteins have its N-terminus ∼150 or ∼230 residues away from the conserved motif F lysine or motif A aspartic acid (corresponding to the yellow and green bars in Figure 1), respectively, while the residue distances between C-terminal boundary of the catalytic module and the conserved motif C aspartic acid are different (∼130 residues for 3D^pol^ vs. ∼250 residues for NS5B). The thumb domain usually starts from 50–60 residues after the XGDD sequence and ends at the C-terminal boundary of the catalytic module. The primer-dependent 3D^pol^ contains four helices in the thumb, while the *de novo* NS5B contains seven. If compared to 3D^pol^, NS5B has one insertion between the third and fourth helices, three extra helices after the fourth helix, and a C-terminal extension (Hansen et al., 1997; Lesburg et al., 1999). It has been suggested that the insertion and the extension together form a priming platform, interacting with the 3′-end of the template and the initiating NTPs to facilitate the *de novo* initiation (Luo et al., 2000; Appleby et al., 2015). In subsequent analyses, we use 150 or 230 residues from motif F lysine or motif A aspartic acid to estimate the N-terminal boundary (corresponding to the yellow and green arrows in Figure 1) and 250 residues from the motif C aspartic acid to estimate the C-terminal boundary (corresponding to the vertical bar on the right hand side in Figure 1) for RdRPs without three-dimensional (3D) structure reported.

## Representative 3D Structures of RdRPs From the Positive-Strand RNA Viruses

**FIGURE 3:**
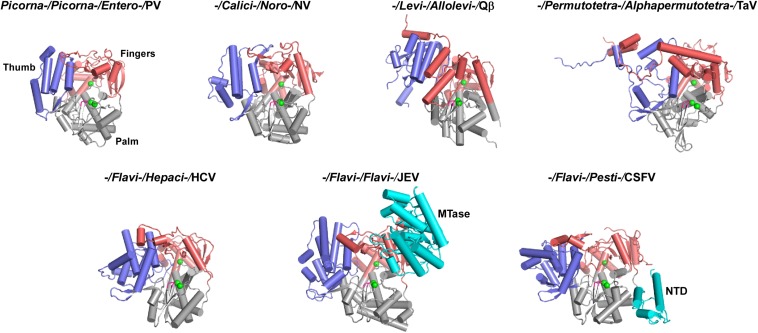
Global views of seven representative RdRPs 3D structures. RdRP structures are shown in cartoon representations. If available, order/family/genus/species assignments are shown on top of each structure. PV, poliovirus, PDB entry 1RA6 (chain A); NV, norovirus, PDB entry 1SH0 (chain A); Qβ, bacteriophage Qβ, PDB entry 3MMP (chain G); TaV, Thosea asigna virus, PDB entry 4XHI (chain A); HCV, hepatitis C virus, PDB entry 1C2P (chain A); JEV, Japanese encephalitis virus, PDB entry 4K6M (chain A); CSFV, classical swine fever virus, PDB entry 5YF5 (chain A). Coloring scheme: RdRP palm in gray, thumb in blue, fingers in pink, and signature sequence XGDD in magenta. The α-carbon atom of the three absolutely conserved amino acid residues (labeled by asterisks in Figure 2) are shown as green spheres. The N-terminal additional regions, if present, are shown in cyan.

Three-dimensional RdRPs structures have been reported for about 20 positive-strand RNA virus species (Hansen et al., 1997; Lesburg et al., 1999; Ng et al., 2002, 2004; Choi et al., 2004; Ferrer-Orta et al., 2004; Love et al., 2004; Fullerton et al., 2007; Malet et al., 2007; Yap et al., 2007; Campagnola et al., 2008; Takeshita and Tomita, 2010; Wu et al., 2010; Lu and Gong, 2013; Vives-Adrian et al., 2014; Ferrero et al., 2015; Bi et al., 2017; Upadhyay et al., 2017; Wang et al., 2017; Liu et al., 2018). However, these species only cover five virus families (*Picornaviridae*, *Caliciviridae*, *Flaviviridae*, *Leviviridae*, and *Permutotetraviridae*) (Figures 1, 3). Among structure-available RdRPs in each virus family, only the RdRPs from the *Flaviviridae* exhibit apparent global structure diversity and have three distinct structural forms. Therefore, a total of seven RdRP structures, including three from the *Flaviviridae*, were chosen as representatives for a schematic illustration of RdRP global structure diversity in positive-strand RNA viruses (Figure 3). Among these seven structures, five of them do not contain functional regions beyond the polymerase catalytic module (Lesburg et al., 1999; Ng et al., 2002; Thompson and Peersen, 2004; Takeshita and Tomita, 2010; Ferrero et al., 2015), although the full-length Thosea asigna virus (TaV) RdRP does contain a large C-terminal region (discussed below). While the structural details are quite different, all these structures are composed of the palm, fingers, and thumb domains and share similar global architecture. The flavivirus NS5 and the pestivirus NS5B, both from the *Flaviviridae* family, are the only RdRP structures contain additional functional regions (Lu and Gong, 2013; Liu et al., 2018). The N-terminal ∼260 residues of the flavivirus NS5 is a methyltransferase (MTase) that participates in the 5′-capping process of the virus RNA genome (Egloff et al., 2002; Koonin, 1993). Based on full-length NS5 crystal structures solved in Japanese encephalitis virus (JEV), dengue virus (DENV), and Zika virus (ZIKV), the MTase adopts the Rossmann fold and interacts with the RdRP fingers domain intra-molecularly in two different modes, one represented by the JEV and ZIKV structures and the other represented by the DENV structures (Lu and Gong, 2013; Upadhyay et al., 2017; Zhao et al., 2015). The N-terminal ∼90 residues of the pestivirus NS5B folds into a small α/β globular domain (namely NTD). The NTD forms intra-molecular interactions with the RdRP palm domain (Li et al., 2018; Liu et al., 2018). Collectively, only a couple of representative RdRP structural forms contain functional regions beyond the RdRP catalytic module. However, the following primary structure analysis suggest that numerous representative RdRPs may have functional regions fused to the catalytic module, in particular to the N-terminus.

## The Diversity of the RdRP Primary Structure in the Positive-Strand RNA Viruses

With the assignment of the key motifs and the size estimation between these motifs and boundaries of the catalytic module, we are able to predict whether functional regions exist at both N- and C-terminal sides of the catalytic module and the approximate size of these regions using the aforementioned criterion, in particular, for those RdRPs with defined boundaries. Interestingly, most of these additional functional regions with an estimated size of 100 residues or larger were found at the N-terminal side of the catalytic module (14 out of 30 families with defined N-terminus), while much fewer showed up at the C-terminal side (4 out of 37 families with defined C-terminus) (Figure 1). The preference of “recruiting” N-terminal regions may be related to two factors. Firstly, among all 46 virus families surveyed, 22 families have the RdRP coding region located at the 3′-end of a polyprotein open reading frame (ORF), while 16 families have the RdRP coding region in the middle of an ORF and 8 families have the RdRP coding region as an independent ORF. Secondly, the *de novo* RdRPs tend to have important initiation elements located at its C-terminus (e.g., the aforementioned priming platform components in HCV NS5B). Both of these factors could reduce the opportunity for RdRPs to recruit additional regions to their C-termini during their evolution. Among these RdRPs containing additional functional regions, some of them have long drawn attentions in the related field but without much advances in structure and related functional characterization of the additional regions. The *Coronaviridae* nsp12 and *Arteriviridae* nsp9 have a ∼200–400 residue N-terminal region with both the structure and function remaining elusive (Gorbalenya et al., 1989; Xu et al., 2003; Beerens et al., 2007; te Velthuis et al., 2010). The *Coronaviridae* nsp8, which can form a supercomplex with nsp7 (each protein contributes eight copies) (Zhai et al., 2005), may have RNA-dependent primase activities (Imbert et al., 2006), and were shown to facilitate the nsp12 RdRP activities along with nsp7 (Subissi et al., 2014b). However, whether and how the N-terminal region of nsp12 might participate in RdRP catalysis or interactions with nsp7/nsp8 remain unclarified. The *Permutotetraviridae* RdRP that contains a ∼600-residue C-terminal region only has the catalytic module structure solved (Ferrero et al., 2015). The nearly 2000-residue *Alphatetraviridae* RdRP has an MTase and a helicase in its N-terminal region and a ∼500-residue C-terminal region with unknown function (Gorbalenya et al., 2002). The *Kitaviridae* RdRP has a helicase in its N-terminal region (Quito-Avila et al., 2013). The *Togaviridae* nsP4 protein has a ∼150-residue N-terminal region that may interact with other viral replication proteins (Lemm et al., 1990; Tomar et al., 2006). The N-terminal ∼300–350 residues of the *Tombusviridae* and *Carmotetraviridae* RdRPs can be produced as proteins (named P33 and P40, respectively) due to a UAG stop codon within the RdRP ORF (Kidmose et al., 2010; Walter et al., 2010). However, the function of these N-terminal regions either as individual proteins or as portions of the RdRP proteins is unknown. Solving the 3D structures of these RdRPs, in particular in their full-length form, is essential to the understanding of these RdRPs. Aside from these RdRPs with relatively large additional regions, some RdRPs with small additional regions may have evolved important functions as well. In the *Flaviviridae* family RdRPs, the hepacivirus and pestivirus NS5B proteins have a 21–24 residue hydrophobic membrane anchor at their C-termini, facilitating its involvement in the replication complex that is located in membranous vesicles derived from endoplasmic reticulum (ER) (Lai et al., 1999; Schmidt-Mende et al., 2001; Appel et al., 2006; Romero-Brey and Bartenschlager, 2014). The ∼90-residue NTD of the pestivirus NS5B modulates the fidelity of RNA synthesis through its intra-molecular interactions with the RdRP palm domain (Liu et al., 2018). Therefore, it will also be quite interesting to dissect the mechanisms involving small but potentially functional regions that may not be readily predicted in our boundary analysis.

## Discussion

Viral RdRPs represent a unique nucleic acid polymerase class of and is the only class that does not involve DNA in any stages of the synthesis. To preserve their RNA template and ribonucleotide triphosphate (rNTP) substrate specificity, the seven catalytic motifs are the central segments to preserve during virus evolution. While we have mainly focused on the diversity and variations beyond the catalytic modules, attentions may also be drawn to the variations within the catalytic module but excluding the catalytic motifs. For the representative RdRPs that we surveyed in this study, the spacing between certain catalytic motifs could vary to a great extent. For example, the residue distance between the conserved motif B serine and the motif C aspartic acid in these RdRPs ranges from 30 to 94, corresponding to a motif spacing of 12–76 residues (Figures 1, 2). We hypothesize that such regions, if located at or near the RdRP protein surface, may have been utilized by the positive-strand RNA viruses as evolutionary “hot spot,” in particular in their host adaptation processes. Further investigations are needed to test this hypothesis.

Although we tried to survey the positive-strand RNA virus RdRPs in a comprehensive manner, there are still several limitations in our analyses. Firstly, the virus species that have not been assigned at the virus family level are not included in our analyses. Secondly, some virus families contain a large number of virus genera (e.g., 47 in *Picornaviridae* and 14 in *Tombusviridae*), suggesting high-level of genome and RdRP diversity within individual families. Moreover, RdRP primary structure diversity and genome-level diversity may not be consistent. The *Flaviviridae* is such an example with only four genera but three drastically different primary RdRP structures. Therefore, choosing one representative RdRP for each family (with the *Flaviviridae* as the only exception) may not be sufficient and ideal. Thirdly, the boundary estimation of the catalytic module is only a crude assessment. For example, the distance between the N-terminal boundary of the TaV RdRP catalytic module to the conserved motif F lysine is at least 100 residues longer than estimated distance using the 150-residue criterion used in our analyses (Ferrero et al., 2015).

In summary, we collected representative RdRPs encoded by positive-strand RNA viruses mainly at the level of virus family. By locating highly conserved residues within catalytic motifs within the RdRP ORF and by referencing structural and functional information of RdRPs in the literature, we tried to estimate the boundaries of the RdRP catalytic module in the full-length RdRP. Numerous regions beyond the RdRP catalytic module exist and many of them have either structure, or function, or both to be determined. Collectively, the global structure and regulatory functions related to regions beyond the catalytic module of the positive-strand RNA virus polymerases are quite diverse, and the current knowledge of these proteins is limited to only a few virus families. One purpose of our analyses is to provide a general guideline for researchers interested in these RdRP proteins and related viral systems to selectively or systematically investigate RdRPs with representative features. The global view of the positive-strand RNA virus RdRPs will continue to evolve with new virus species assigned, new structures determined, new functional regions identified, and new mechanisms dissected.

## Author Contributions

Both authors surveyed the literature, analyzed the data, and wrote the manuscript.

## Conflict of Interest Statement

The authors declare that the research was conducted in the absence of any commercial or financial relationships that could be construed as a potential conflict of interest.

## Footnotes

1 http://www.ictv.org

2 https://www.ncbi.nlm.nih.gov
